# Bulk Acoustic Wave Resonance Characteristics of PMN-PT Orthorhombic Crystal Plates Excited by Lateral Electric Fields

**DOI:** 10.3390/mi16050600

**Published:** 2025-05-21

**Authors:** Boyue Su, Yujie Zhang, Feng Yu, Pengfei Kang, Tingfeng Ma, Peng Li, Zhenghua Qian, Iren Kuznetsova, Vladimir Kolesov

**Affiliations:** 1 Zhejiang-Italy Joint Lab for Smart Materials and Advanced Structures, School of Mechanical Engineering and Mechanics, Ningbo University, Ningbo 315211, China; 2211090027@nbu.edu.cn (B.S.); 2311090044@nbu.edu.cn (Y.Z.); 2211090119@nbu.edu.cn (P.K.); 2 Ningbo Liance Smart Technology Co., Ltd., Ningbo 315211, China; nblcs_yufeng@126.com; 3 The State Key Laboratory of Mechanics and Control of Mechanical Structures, College of Aerospace Engineering, Nanjing University of Aeronautics and Astronautics, Nanjing 210016, China; lipeng_mech@nuaa.edu.cn (P.L.); qianzh@nuaa.edu.cn (Z.Q.); 4 Kotelnikov Institute of Radio Engineering and Electronics of RAS, Moscow 125009, Russia; kuziren@yandex.ru (I.K.); kvv@cplire.ru (V.K.)

**Keywords:** PMN-PT relaxor ferroelectric single crystal, lateral field excitation, high frequency resonance, bulk acoustic wave sensor

## Abstract

For relaxor ferroelectric single crystal (1 − x)Pb(Mg_1/3_Nb_2/3_)O_3_ − xPbTiO_3_ (PMN-PT), through reasonable component regulation and electric field polarization, an orthogonal mm^2^ point group structure can be obtained, which has high piezoelectric constants and is, therefore, a desired substrate material for lateral-field-excited (LFE) bulk acoustic wave (BAW) devices. In this work, acoustic wave resonance characteristics of (*zxt*) 45° PMN-PT BAW devices with LFE are investigated. Firstly, Mindlin first-order plate theory is used to obtain vibration governing equations of orthorhombic crystals excited by a lateral electric field. By analyzing the electrically forced vibrations of the finite plate, the basic vibration characteristics, such as motional capacitance, resonant frequency, and mode shape are obtained, and influences of different electrode parameters on resonance characteristics of the device are investigated. In addition, the effects of the structure parameters on the mass sensitivity of the devices are analyzed and further verified by FEM simulations. The model presented in this study can be conveniently used to optimize the structural parameters of LFE bulk acoustic wave devices based on orthorhombic crystals, which is crucial to obtain good resonance characteristics. The results provide an important basis for the design of LFE bulk acoustic wave resonators and sensors by using PMN-PT orthorhombic crystals.

## 1. Introduction

In recent years, bulk acoustic wave (BAW) devices have attracted much attentions due to high resonance quality factors and high sensitivity to variations in load, which leads to their widespread application in various areas, including resonators, filters, and chemical and biological sensors [1,2,3,4,5].

Conventional BAW devices employ thickness field excitation (TFE) [6], where the electrodes are plated on the top and bottom surfaces of the crystal plate and the electric field is directed along the thickness direction. Lateral field excitation (LFE), where the electrodes are plated on the same side of the crystal plate, shows obvious advantages compared to TFE devices [7,8,9], namely, by varying the direction of the electric field of the LFE, unwanted parasitic modes can be eliminated. In addition, when LFE devices are used for biochemical sensing, by allowing the electrode-free surface to contact with the liquid, the corrosion of the electrodes from the analytes can be reduced, and the lifetime of the device can be improved significantly [10,11,12].

For relaxor ferroelectric single crystal (1−x)Pb(Mg_1/3_Nb_2/3_)O_3_-xPbTiO_3_ (PMN-PT), an orthogonal mm2 point group structure can be obtained through reasonable component regulation and electric field polarization [13,14,15], which has high piezoelectric constants and is, therefore, a desired substrate material for LFE BAW devices. Due to its excellent piezoelectric properties, relaxed ferroelectric single crystals showed widespread application prospects [16,17,18,19,20,21]. H.T. Wong investigated the vibration characteristics of disc-type PMN-PT single-crystal resonators and showed that partial electrodes can effectively confine the main vibration modes to the electrode region [22]. Kyungrim Kim investigated the effect of surface loading on PMN-PT single crystal piezoelectric resonators and found that the electrical impedance of surface shear mode piezoelectric single crystal resonators is more sensitive to surface loadings [23]. However, the resonance characteristics of the relaxed ferroelectric single crystal PMN-PT excited by lateral electric fields are still unclear. Due to the high piezoelectric coupling relationship of the relaxed ferroelectric single crystals, their electric fields and displacement distributions are more complex and the resonance characteristics need to be clarified.

In this paper, Mindlin first-order plate theory is used to investigate the TT_3_ and E_3_ coupled vibrations of the PMN-PT single crystal plate under lateral field excitation. The resonance characteristics of free and forced vibrations of the device are analyzed and, based on this analysis, the influences of structure parameters (electrode width and electrode gap) on the resonance frequency, vibration strength, and mass sensitivity are revealed, which are of great significance to the sensing characteristics of the device. The finite element method is used to validate the theoretical results.

## 2. Model

**Figure 1:**
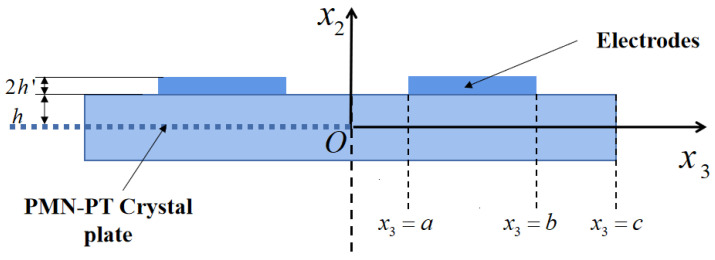
A PMN-PT crystal plate under lateral field excitation.

Consider a rectangular (*zxt*) 45° PMN-PT crystal plate as shown in Figure 1. The thickness, length, and mass density of the crystal plate are 2*h*, 2*c,* and *ρ*, respectively. The crystal axes *x*, *y,* and *z* correspond respectively to x_3_, x_1,_ and x_2_ axes in Figure 1. The crystal plate is infinitely long along the x_1_ axes and symmetric about x_3_ = 0. *a* < |*x*_3_| < *b* is the electrode region, and the thickness and density of the electrodes are 2*h*′ and *ρ*′, respectively. The driven voltage ±Vexp(iωt) is applied to the electrodes, and the electric field *E*_1_ (*x*_3_,*t*) is produced on the region of |*x*_3_| < *a*.

The Mindlin plate equations for orthogonal piezoelectric crystals have slightly different forms for non-electrode and electrode regions. In the non-electrode region of the plate, for thickness-twist and extension motions, the displacements and electric potential fields are approximated as follows [24]

(1) $$u_{3}≅u_{3}^{\left(0\right)}(x_{3},t),\;\;u_{2}≅0,\;\;u_{1}≅x_{2}u_{1}^{\left(1\right)}(x_{3},t),\;\;\;\phi ≅\phi^{\left(0\right)}(x_{1},t),\;\;\;$$

where $u_{1}^{\left(1\right)}(x_{3},t)$ is the thickness twist strain (m), $u_{3}^{\left(0\right)}(x_{3},t)$ is the tensile strain (m) and $\phi^{\left(0\right)}$ is the electrical potential (V). The controlling equations for $u_{1}^{\left(1\right)}$, $u_{3}^{\left(0\right)}$, and $\phi^{\left(0\right)}$ are as follows:

(2) $$\begin{array}{l} T_{3,3}^{\left(0\right)}=2h\rho {\ddot{u}}_{3}^{\left(0\right)}\\ T_{5,3}^{\left(1\right)}-T_{6}^{\left(0\right)}=\frac{2h^{3}}{3}\rho {\ddot{u}}_{1}^{\left(1\right)}\\ D_{3,3}^{\left(0\right)}=0 \end{array}$$

In Equation (2), $T_{3}^{\left(0\right)},T_{5}^{\left(1\right)}\;\mathrm{and}\;D_{3}^{\left(0\right)}\;$ can be obtained from the following constitutive equations, namely

(3) $$\begin{array}{l} T_{3}^{\left(0\right)}=2h({\bar{c}}_{33}u_{3,3}^{\left(0\right)}+k_{1}{\bar{c}}_{36}u_{1}^{\left(1\right)}+{\bar{e}}_{33}\phi_{,3}^{\left(0\right)}),\\ T_{6}^{\left(0\right)}=2h(k_{1}{\bar{c}}_{63}u_{3,3}^{\left(0\right)}+k_{1}^{2}{\bar{c}}_{66}u_{1}+k_{1}{\bar{e}}_{36}\phi_{,3}^{\left(0\right)}),\\ T_{5}^{\left(1\right)}=\frac{2h^{3}}{3}\gamma_{55}u_{1,3}^{\left(1\right)},\\ D_{3}^{\left(0\right)}=2h({\bar{e}}_{33}u_{3,3}^{\left(0\right)}+k_{1}{\bar{e}}_{36}u_{1}^{\left(1\right)}-\varepsilon_{33}\phi_{,3}^{\left(0\right)}), \end{array}$$

where

(4) $$\begin{array}{l} {\bar{c}}_{33}=c_{33}-\frac{c_{32}c_{22}}{c_{22}},\;\;\;\;\;\;\;\;{\bar{c}}_{36}=k_{1}(c_{36}-\frac{c_{32}c_{26}}{c_{22}}),\\ {\bar{e}}_{33}=e_{33}-\frac{e_{32}c_{32}}{c_{22}},\;\;\;\;\;\;\;\;{\bar{e}}_{36}=k_{1}(e_{36}-\frac{e_{32}c_{62}}{c_{22}}),\\ \gamma_{55}=c_{55}-\frac{c_{56}^{2}}{c_{66}}. \end{array}$$

In Equation (4), $c_{pq}(=c_{pq}^{E})$, $\varepsilon_{ij}$, and $e_{iq}(=e_{ip}^{E})$ are the elastic constant, the dielectric constant, and the piezoelectric constant, respectively. The substitution of Equation (3) into Equation (2) yields

(5) $$\begin{array}{l} {\bar{c}}_{33}u_{3,3}^{\left(0\right)}+k_{1}{\bar{c}}_{36}u_{1}^{\left(1\right)}+{\bar{e}}_{33}\phi_{,3}^{\left(0\right)}=\rho {\ddot{u}}_{3}^{\left(0\right)},\\ \gamma_{55}u_{1,33}^{\left(1\right)}-\;\frac{3}{h^{2}}(k_{1}{\bar{c}}_{63}u_{3,3}^{\left(0\right)}+k_{1}^{2}{\bar{c}}_{66}u_{1}+k_{1}{\bar{e}}_{36}\phi_{,3}^{\left(0\right)})=\rho {\ddot{u}}_{1}^{\left(1\right)},\\ {\bar{e}}_{33}u_{3,3}^{\left(0\right)}+k_{1}{\bar{e}}_{36}u_{1}^{\left(1\right)}-\varepsilon_{33}\phi_{,3}^{\left(0\right)}=0. \end{array}$$

For the electrode region, since the potential $\phi^{\left(1\right)}$ is a constant (and may still depend on time), and considering the mass ratio *R* of the electrodes, the motion governing equation for the electrode region is

(6) $$\begin{array}{l} T_{3,3}^{\left(0\right)}=2h\rho (1+R){\ddot{u}}_{3}^{\left(0\right)},\\ T_{5,3}^{\left(1\right)}-T_{6}^{\left(0\right)}=\frac{2h^{3}}{3}\rho (1+3R){\ddot{u}}_{1}^{\left(1\right)},\;\;\;\;\;\;\;\;\;\;\;\; \end{array}$$

where $R=\rho^{\prime }h^{\prime }/(\rho h)<<1$ is the electrode/plate mass ratio. The constitutive equation of the electrode region is

(7) $$\begin{array}{l} T_{3}^{\left(0\right)}=2h({\bar{c}}_{33}u_{3,3}^{\left(0\right)}+{\bar{k}}_{1}{\bar{c}}_{36}u_{1}^{\left(1\right)}),\\ T_{6}^{\left(0\right)}=2h({\bar{k}}_{1}{\bar{c}}_{63}u_{3,3}^{\left(0\right)}+{\bar{k}}_{1}^{2}{\bar{c}}_{66}u_{1}),\\ T_{5}^{\left(0\right)}=\frac{2h^{3}}{3}\gamma_{55}u_{1,3}^{\left(1\right)},\;\; \end{array}$$

where ${\bar{k}}_{1}^{2}=k_{1}^{2}(1+R)$; the substitution of Equation (7) into Equation (6) yields

(8) $$\begin{array}{l} {\bar{c}}_{33}u_{3,3}^{\left(0\right)}+{\bar{k}}_{1}{\bar{c}}_{36}u_{1}^{\left(1\right)}=\rho (1+R){\ddot{u}}_{3}^{\left(0\right)},\\ \gamma_{55}u_{1,33}^{\left(1\right)}-\;\frac{3}{h^{2}}({\bar{k}}_{1}{\bar{c}}_{63}u_{3,3}^{\left(0\right)}+k_{1}^{2}{\bar{c}}_{66}u_{1})=\rho (1+3R){\ddot{u}}_{1}^{\left(1\right)}. \end{array}$$

In this work, the high-frequency vibration of the device is analyzed by using the Mindlin plate theory (a two-dimensional model). The principle of Mindlin plate theory involves expanding the displacement and electric potential into power series along the thickness direction and truncating the equations by neglecting higher-order displacement and potential terms. By retaining only the leading terms of the series, the three-dimensional governing equations are reduced to two-dimensional ones. The device investigated in this work is a thin plate with a thickness of 0.143 mm and, therefore, the associated frequency deviation resulting from higher-order modes is negligible.

## 3. Electrically Forced Vibrations of the Finite Plate

Since the finite plate is symmetric about x_3_ = 0 and the applied voltage is antisymmetric, the electromechanical coupling field is symmetric or antisymmetric about x_3_ = 0. In this work, only the right half of the crystal plate is considered. On the right half of the plate, only a part is covered by the electrode; thus, the right half of the plate needs to be divided into three parts (Figure 1), namely the central non-electrode region, the electrode region, and the external non-electrode region.

### 3.1. Central Non-Electrode Area $0<x_{3}<a$

We consider the displacement and potential in the following form

(9) $$\begin{array}{l} u_{3}^{\left(0\right)}=A_{1}\sin(\xi x_{3})\exp(i\omega t),\\ u_{1}^{\left(1\right)}=A_{2}\cos(\xi x_{3})\exp(i\omega t),\\ \phi^{\left(0\right)}=A_{3}\sin(\xi x_{3})\exp(i\omega t), \end{array}$$

where $A_{1}-A_{3}$ are constants to be determined. Substituting Equation (9) into Equation (5) yields a third-order linear equation of $A_{1}-A_{3}$. The determinant of the coefficient matrix must be zero for nontrivial solutions, which yields a polynomial equation of degree four of $\xi^{2}$. Solving this polynomial gives three solutions for the wave number ${\left(\xi^{\left(m\right)}\right)}^{2}$ (m = 1–2), two non-zero solutions, and one zero solution. The displacements and the electric potential take the following solution forms.

(10) $$\left\{\begin{array}{l} u_{3}^{\left(0\right)}\\ u_{1}^{\left(1\right)}\\ \phi^{\left(0\right)} \end{array}\right\}=\sum_{m=1}^{2}{\bar{C}}^{\left(m\right)}\left\{\begin{array}{l} \beta_{1}^{\left(m\right)}\sin(\xi^{\left(m\right)}x_{3})\\ \beta_{2}^{\left(m\right)}\cos(\xi^{\left(m\right)}x_{3})\\ \beta_{3}^{\left(m\right)}\sin(\xi^{\left(m\right)}x_{3}) \end{array}\right\}+{\bar{C}}^{\left(3\right)}\left\{\begin{array}{l} 0\\ B_{1}\\ x_{3} \end{array}\right\},$$

where $C^{\left(1\right)}-C^{\left(3\right)}$ are unknown constants, $\beta_{\rho }^{\left(m\right)}$ is the ratio of $A_{1}-A_{3}$, $\beta_{3}^{\left(m\right)}=1$, and

(11) $$B_{1}=\frac{-k_{1}{\bar{e}}_{36}}{k_{1}^{2}{\bar{c}}_{66}-\pi^{2}\Omega^{2}c_{66}/12}.$$

### 3.2. Electrode Area $a<x_{3}<b$

In the electrode region, $\phi^{\left(0\right)}$ is known, and the displacements take the form of

(12) $$\begin{array}{l} u_{3}^{\left(0\right)}=A_{1}\exp(i\bar{\xi }x_{3})\exp(i\omega t),\\ u_{1}^{\left(1\right)}=A_{2}\exp(i\bar{\xi }x_{3})\exp(i\omega t). \end{array}$$

Substituting Equation (12) into Equation (8) yields a second-order linear equation of $A_{1}-A_{2}$. To obtain non-zero solutions of $A_{1}-A_{2}$, the determinant of its coefficients must be zero, which gives a second-order polynomial of ${\bar{\xi }}^{2}$. Solving this polynomial gives four solutions for the wave number ${\left({\bar{\xi }}^{\left(m\right)}\right)}^{2}$ (m = 1–2). The displacements and the electric potentials take the following solution forms.

(13) $$\left\{\begin{array}{l} u_{3}^{\left(0\right)}\\ u_{1}^{\left(1\right)} \end{array}\right\}=\sum_{m=1}^{4}{\bar{C}}^{\left(m\right)}\left\{\begin{array}{l} \beta_{1}^{\left(m\right)}\exp(i{\bar{\xi }}^{\left(m\right)}x_{3})\\ \beta_{2}^{\left(m\right)}\exp(i{\bar{\xi }}^{\left(m\right)}x_{3}) \end{array}\right\},$$

where ${\bar{C}}^{\left(1\right)}-{\bar{C}}^{\left(4\right)}$ are constants to be determined.

### 3.3. External Non-Electrode Area $b<x_{3}<c$

The displacements and potential are assumed to be

(14) $$\begin{array}{l} u_{3}^{\left(0\right)}=A_{1}\exp(i\tilde{\xi }x_{3})\exp(i\omega t),\\ u_{1}^{\left(1\right)}=A_{2}\exp(i\tilde{\xi }x_{3})\exp(i\omega t),\\ \phi^{\left(0\right)}=A_{3}\exp(i\tilde{\xi }x_{3})\exp(i\omega t). \end{array}$$

Substituting Equation (14) into Equation (5) yields a third-order linear equation of $A_{1}-A_{3}$. To obtain non-zero solutions of $A_{1}-A_{3}$, the determinant of its coefficients must be zero, which gives a third-order polynomial of ${\tilde{\xi }}^{2}$. Solving this polynomial gives six solutions for the wave number ${\left({\tilde{\xi }}^{\left(m\right)}\right)}^{2}$ (m = 1–2), four of which are non-zero and two which are zero. The displacements, electric potentials are assumed with the following solution forms.

(15) $$\left\{\begin{array}{l} u_{3}^{\left(0\right)}\\ u_{1}^{\left(1\right)}\\ \phi^{\left(0\right)} \end{array}\right\}=\sum_{m=1}^{4}{\tilde{C}}^{\left(m\right)}\left\{\begin{array}{l} {\bar{\beta }}_{1}^{\left(m\right)}\exp(i{\tilde{\xi }}^{\left(m\right)}x_{3})\\ {\bar{\beta }}_{2}^{\left(m\right)}\exp(i{\tilde{\xi }}^{\left(m\right)}x_{3})\\ {\bar{\beta }}_{3}^{\left(m\right)}\exp(i{\tilde{\xi }}^{\left(m\right)}x_{3}) \end{array}\right\}+{\tilde{C}}^{\left(5\right)}\left\{\begin{array}{l} 0\\ {\bar{B}}_{1}\\ x_{3} \end{array}\right\}+{\tilde{C}}^{\left(6\right)}\left\{\begin{array}{l} 0\\ 0\\ 1 \end{array}\right\},$$

where ${\tilde{C}}^{\left(1\right)}-{\tilde{C}}^{\left(6\right)}$ are constants to be determined and ${\bar{B}}_{1}=-B_{1}$.

### 3.4. Boundary and Continuity Conditions

The boundary conditions and continuity conditions of the resonance system are shown below.

The continuity conditions at $x_{1}=a$ are

(16) $$\begin{array}{l} u_{3}^{\left(0\right)}(x_{3}=a^{-})=u_{3}^{\left(0\right)}(x_{3}=a^{+}),\\ u_{1}^{\left(1\right)}(x_{3}=a^{-})=u_{1}^{\left(1\right)}(x_{3}=a^{+}),\\ T_{3}^{\left(0\right)}(x_{3}=a^{-})=T_{3}^{\left(0\right)}(x_{3}=a^{+}),\\ T_{5}^{\left(1\right)}(x_{3}=a^{-})=T_{5}^{\left(1\right)}(x_{3}=a^{+}),\\ \phi^{\left(0\right)}(x_{3}=a^{-})=V\exp(i\omega t). \end{array}$$

The continuous conditions at $x_{1}=b$ are

(17) $$\begin{array}{l} u_{3}^{\left(0\right)}(x_{3}=b^{-})=u_{3}^{\left(0\right)}(x_{3}=b^{+}),\\ u_{1}^{\left(1\right)}(x_{3}=b^{-})=u_{1}^{\left(1\right)}(x_{3}=b^{+}),\\ T_{3}^{\left(0\right)}(x_{3}=b^{-})=T_{3}^{\left(0\right)}(x_{3}=b^{+}),\\ T_{5}^{\left(1\right)}(x_{3}=b^{-})=T_{5}^{\left(1\right)}(x_{3}=b^{+}),\\ \phi^{\left(0\right)}(x_{3}=b^{+})=V\exp(i\omega t). \end{array}$$

The boundary conditions at $x_{1}=c$ are

(18) $$\begin{array}{l} T_{3}^{\left(0\right)}(x_{1}=c^{-})=0,\\ T_{5}^{\left(1\right)}(x_{1}=c^{-})=0,\\ D_{3}^{\left(0\right)}(x_{1}=c^{-})=0. \end{array}$$

The unknown constants $C^{\left(1\right)}-C^{\left(3\right)}$, ${\bar{C}}^{\left(1\right)}-{\bar{C}}^{\left(2\right)}$ and ${\tilde{C}}^{\left(1\right)}-{\tilde{C}}^{\left(6\right)}$ can be obtained by substituting Equations (9), (12), and (14) into Equations (16)–(18). Once these constants are determined, the displacements and electric potentials of the resonator are also known for crystal plates. Furthermore, the charge $Q_{e}$, the dynamic capacitance C, and the static capacitance $C_{0}$ can be achieved by the following equations.

(19) $$\begin{array}{l} Q_{e}=-D_{3}^{\left(0\right)}(x=a^{-})\cdot 2w,C=\frac{Q_{e}}{2V},\\ C_{0}=\frac{4\varepsilon_{33}hw}{2c}, \end{array}$$

where *w* is half of the width of the crystal plate in the *x*_3_ direction.

### 3.5. Numerical Results and Discussion

In numerical calculations, material parameters of the PMN-PT single crystal with a cut of (*zxt*) 45° are obtained from the literature, as shown in Table 1 [25], and the fundamental frequency of the device is 5 MHz. Taking into account the dissipative damping of the material, the elastic constants are with a complex form, namely $\left(1+iQ^{-1}\right)$, where i is an imaginary number, and Q is a real number with a large value. In this case, *Q* = 10^4^ is taken to represent the total dissipative damping of the material. The size parameters of the crystal plate are 2*h* = 0.14 mm, *a* = 0.36 mm, *b* = 2.44 mm, *c* = 4.89 mm, *w* = 9.77 mm, and *R* = 0.005.

**Figure 2:**
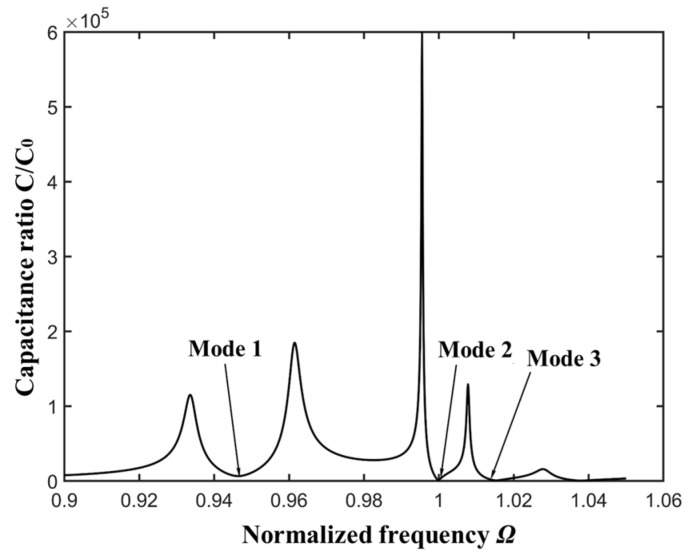
Normalized capacitance (capacitance ratio) versus driving frequency.

**Figure 3:**
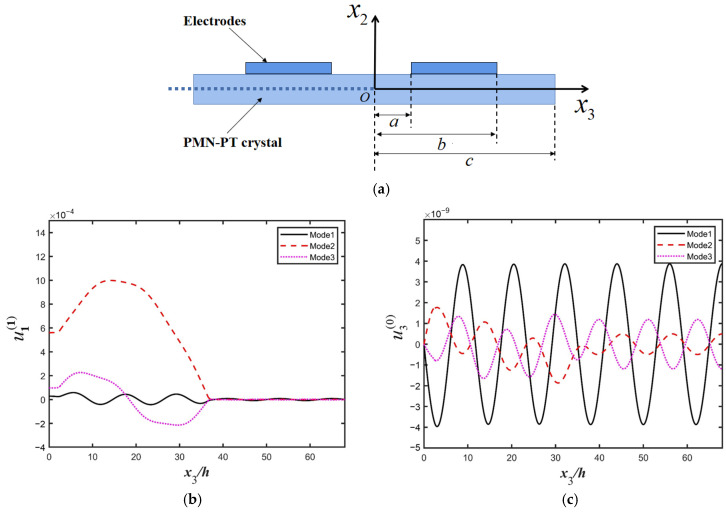
(**a**) Schematic diagram of the device structure, (**b**) thickness-twist strain distribution $\left(u_{1}^{\left(1\right)}\right)$, (**c**) extensional strain distribution $\left(u_{3}^{\left(0\right)}\right)$.

Figure 2 represents the relationship between the absolute value of capacitance ratio |*C*/*C*_0_| and the normalized driving frequency, which is obtained from an electrically forced vibration analysis. The dynamic capacitance, which is directly governed by the driving voltage and the accumulated charge, can be calculated conveniently based on the analysis model of this work. The resonance frequency of TT_3_ mode is slightly below the fundamental frequency $\omega_{0}$ due to the piezoelectric stiffness effect. Three main resonance frequencies Mode 1, Mode 2, and Mode 3 in Figure 2 are 0.947 $\omega_{0}$, 0.998 $\omega_{0}$, 1.01 $\omega_{0}$, respectively. Near mode 2, due to the anti-resonance, a sharp peak appears. The strain curves of thickness-twist ($u_{1}^{\left(1\right)}(x_{3})$) and extension ($u_{3}^{\left(0\right)}(x_{3})$) modes are plotted and shown in Figure 3b and Figure 3c, respectively.

Due to the symmetry, Figure 3 shows strain distributions of half of the crystal plate. As shown in Figure 3b, for Mode 2, thickness-twist strains are mainly concentrated within the electrode region, showing an obvious attenuating in the non-electrode region. Thus, for Model 2, the energy trapping of the thickness-twist mode is good. For Mode l, the strain difference between the electrode and non-electrode regions is not obvious. For Mode 3, the vibration intensity is not strong enough, although it has a certain energy-trapping effect. As shown in Figure 3c, for Mode 2 and Mode 3, the tension strains are weak, while for Mode 1, the tension strains are stronger.

As a whole, Mode 2 presents a good energy-trapping effect for thickness-twist motion and a weak vibration for tension motion; therefore, it is suitable for being used as an ideal operational mode of LFE bulk acoustic wave devices. The strain energy is proportional to the square of the strain. The strain of the thickness-shear mode (main mode) is larger than that of the extensional strain mode by five orders of magnitude, indicating that the device exhibits excellent suppression of parasitic extensional modes and achieves high energy utilization efficiency.

## 4. Influences of Structure Parameters on the Resonance Characteristics of the PMN-PT LFE Device

Finite element method (FEM) simulations are performed using the COMSOL Multiphysics 5.4, employing the Solid Mechanics and Electrostatics modules for multi-physical coupling analysis. In the Solid Mechanics domain, the coordinate system of the piezoelectric material is defined as a rotated coordinate system to obtain a correctly oriented PMN-PT single crystal. For the electrodes covering the crystal plate, conventional meshing approaches significantly reduce accuracy due to their extremely small thickness. To address this, a special treatment is applied: the 3D electrode geometry is simplified into a 2D planar representation, and the additional mass is introduced on the electrode plane to compensate for the loss of thickness-related effects.

**Figure 4:**
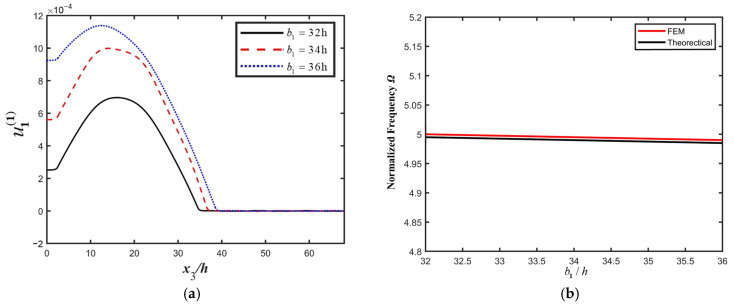
The influence of electrode width *b*_1_ on the resonance characteristics of the PMN-PT LFE device. (**a**) Thickness-twist strain $\left(u_{1}^{\left(1\right)}\right)$ for different electrode widths *b*_1_; (**b**) resonance frequency for different electrode width *b*_1_.

Influences of structure parameters on the resonance characteristics of the PMN-PT LFE device are calculated, and the results are shown in Figure 4. Parameters are set as 2h=0.14 mm, a=0.36 mm, c=9.77 mm, and R=0.005.. Figure 4a shows that as the width of the electrode increases, the vibration intensity increases. A larger electrode width leads to a lower impedance; thus, the energy trapping effect becomes stronger. Additionally, in Figure 4b, it is shown that the resonant frequency of the device decreases slightly with the increasing electrode width. The increase in the electrode width leads to the increase in the electrode mass, and the resonant frequency decreases accordingly, resulting from the mass effect of the BAW device. Considering the actual installation of the device, a certain installation region near the side of the crystal plate needs to be reserved; thus, the width of the electrode cannot be too large—the electrode radius usually should be smaller than 40% of the device radius to ensure proper vibration confinement.

**Figure 5:**
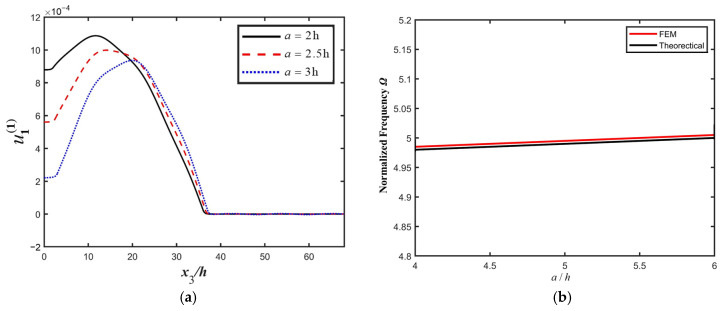
Influences of the electrode gap width value *a* on the resonance characteristics of the PMN-PT LFE device. (**a**) Thickness-twist strain $\left(u_{1}^{\left(1\right)}\right)$ for different electrode gap values *a*, (**b**) device resonance frequency for different electrode gap values *a*.

Figure 5 shows the influences of the electrode gap on resonance characteristics of the PMN-PT LFE device. Structure parameters are set as 2h=0.14 mm, b=2.44 mm, c=9.77 mm, and R=0.005. Figure 5a shows that as the width of the electrode gap increases, the vibration intensity decreases markedly. The larger electrode gap width contributes to higher device vibration impedance; thus, the vibration becomes weaker. Additionally, in Figure 5b, it is shown that the resonance frequency of the device increases slightly with the increase in electrode gap width. The increase in the electrode gap width decreases the electrode mass; thus, the resonance frequency increases accordingly, resulting from the mass effect of the BAW device.

**Figure 6:**
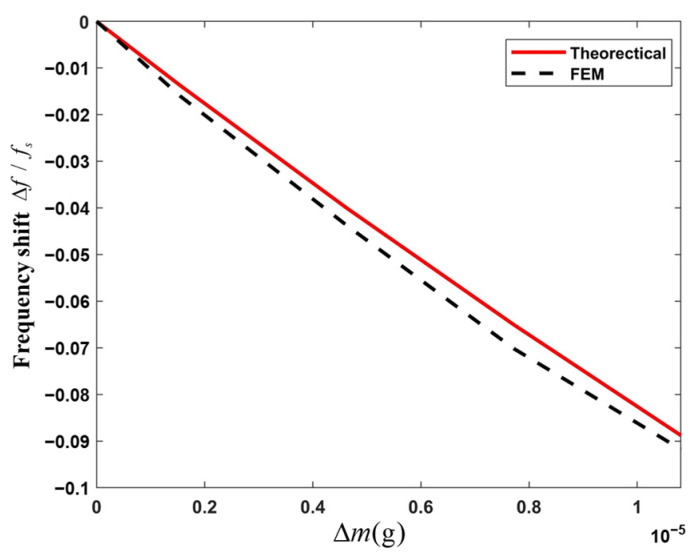
Effects of the added micro-mass on the shift of the resonance frequency.

When a load is applied to the surface of the BAW resonator, the resonant frequency decreases due to the increase in the load mass, which results in a negative frequency shift phenomenon, which is called the mass loading effect. The definition of the frequency shift to reflect the influence of the micro-mass on the resonance frequency of the device [26] is $\Delta f/f_{s}$, where Δf is the shift of the resonance frequency, and $f_{s}$ is the reference resonance frequency. The device parameters are set as R=0.005, b=34h, c=2.5h. The frequency shifts caused by a gradual increase in additional mass on the device is shown in Figure 6. It is shown that the frequency shift decreases linearly with the increase in additional mass. The frequency shifts obtained from the theory are slightly larger than those from the simulation.

**Figure 7:**
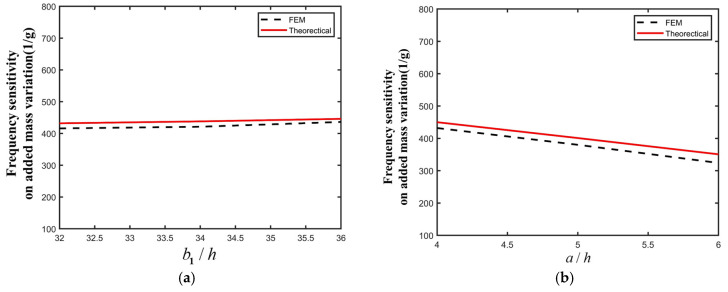
(**a**) Frequency sensitivities corresponding to different electrode widths *b*_1_. (**b**) Frequency sensitivities corresponding to different electrode gap values *a*.

The influence of structure parameters of the PMN-PT crystal plate under LFE on the frequency sensitivity are analyzed, where the frequency sensitivity is the ratio of the change in the frequency shift to the value of the added surface micro-mass. The results are shown in Figure 7. As shown in Figure 7a, the frequency sensitivity gradually increases with the increase in the electrode width *b*_1_. The reason for this phenomenon is that as the electrode width increases, the vibration intensity of the device increases and, thus, the corresponding micro-mass-induced frequency sensitivity is enhanced. In Figure 7b, with the increase in electrode spacing a, the frequency sensitivity gradually decreases. The reason is that as the electrode gap width increases, the intensity of the corresponding lateral electric field gradually weakens, and the vibration intensity of the device gradually decreases, resulting in decreased frequency sensitivity.

In addition to the above, the relationships between resonant frequency, Q factor, electromechanical coupling coefficient, and structural parameters are further supplemented as follows: The resonant frequency of a PMN-PT LFE resonator is primarily determined by the acoustic phase velocity (*v*) and the crystal thickness (2*h*), following the relation $f_{r}=\frac{v}{2h}$. In addition, due to the mass loading effect of bulk acoustic wave (BAW) devices, the added mass of the electrodes can slightly decrease the resonant frequency. The Q-factor refers to the ratio of the total energy stored to the energy dissipated within one cycle. Factors influencing the dissipated energy include acoustic wave scattering caused by internal crystal defects (such as dislocations and impurities) and acoustic wave reflection losses due to the roughness at the electrode-crystal interface. Therefore, to enhance the Q-factor of the resonator, the crystal surface is usually polished. For LFE piezoelectric crystal resonators, the electromechanical coupling coefficient is a key parameter reflecting the efficiency of energy conversion between electrical and mechanical forms. It is closely related to the following factors: The piezoelectric constant of the material directly determines the coupling strength between the electric field and mechanical strain. For PMN-PT, the high piezoelectric constant is beneficial for enhancing the electromechanical coupling coefficient. Additionally, the crystal symmetry and polarization direction play a role—for PMN-PT, an orthogonal mm^2^ point group structure can be achieved through proper component regulation and electric field polarization (applying an electric field along the [110] direction), which results in high piezoelectric constants. The conductivity of the electrodes affects the ohmic losses, which in turn indirectly influences the effective coupling coefficient. Therefore, in this study, gold electrodes with good conductivity are selected. However, the increase in electrode thickness adds inertia, which slightly reduces the electromechanical coupling coefficient.

This work focuses on the linear vibration behavior of the device. When operating in the linear regime (i.e., under a relatively low driving voltage), the vibration displacement is proportional to the driving voltage $V_{0}$), following the relation $u_{\max}\propto V_{0}$, indicating a linear electromechanical response. In this case, the resonant frequency remains stable, and the Q-factor is high. However, when the device operates in the nonlinear regime (i.e., under a sufficiently high driving voltage), the strain in the piezoelectric material approaches its saturation limit, causing $u_{\max}$ to deviate from the linear increasing with respect to $V_{0}$, and higher-order harmonics may be generated. Additionally, increased electrode resistance heating and enhanced acoustic wave scattering at a high driving voltage lead to the degradation of the Q-factor.

## 5. Conclusions

In this work, acoustic wave resonance characteristics of LFE BAW devices based on orthorhombic crystals PMN-PT are modeled and analyzed. Mindlin first-order plate theory is used to obtain vibration governing equations of orthorhombic crystals excited by a lateral electric field. By analyzing electrically forced vibrations of the finite PMN-PT plate, vibration characteristics such as motional capacitance, resonant frequency, and displacement distribution are obtained, and influences of different electrode parameters on the resonance characteristics of the device are examined. In addition, the effects of the structure parameters on the mass sensitivity of the devices are analyzed, which are further verified by FEM simulations. By using the model in this study, for LFE bulk acoustic wave devices based on orthorhombic crystals, suitable structural parameters can be obtained conveniently, which is vital for obtaining good resonance characteristics and sensitivities of the device. The results can provide an important basis for the design of LFE bulk acoustic wave resonators and sensors based on PMN-PT orthorhombic crystals.

## Figures and Tables

**Table 1:** Main parameters for (*zxt*) 45° PMN-PT crystal.

Parameter	Symbol	Numerical Values
Density	ρ	8120 kg/m^3^
Young’s modulus	$Y_{33}$	2.2 × 10^10^ N/m^2^
Poisson’s ratio	v	0.34
Dielectric constant	$\varepsilon_{33}/\varepsilon_{0}$	8783
Piezoelectric constant	$d_{33}$	1913 pc/N
Electromechanical coupling Coefficient	$k_{33}$	0.92
Quality factor	Q	150

## Data Availability

The original contributions presented in the study are included in the article, further inquiries can be directed to the corresponding author.
